# A 10-Year Population Based Study of ‘Opt-Out’ HIV Testing of Tuberculosis Patients in Alberta, Canada: National Implications

**DOI:** 10.1371/journal.pone.0098993

**Published:** 2014-06-09

**Authors:** Richard Long, Selvanayagam Niruban, Courtney Heffernan, Ryan Cooper, Dina Fisher, Rabia Ahmed, Mary Lou Egedahl, Rhonda Fur

**Affiliations:** 1 Department of Medicine, University of Alberta, Edmonton, Alberta, Canada; 2 Department of Public Health Sciences, University of Alberta, Edmonton, Alberta, Canada; 3 Department of Medicine, University of Calgary, Calgary, Alberta, Canada; 4 Alberta Health Services, Alberta, Canada; World Health Organization, Switzerland

## Abstract

**Introduction:**

Compliance with the recommendation that all tuberculosis (TB) patients be tested for human immunodeficiency virus (HIV) has not yet been achieved in Canada or globally.

**Methods:**

The experience of “opt-out” HIV testing of TB patients in the Province of Alberta, Canada is described over a 10-year period, 2003–2012. Testing rates are reported before and after the introduction of the “opt-out” approach. Risk factors for HIV seropositivity are described and demographic, clinical and laboratory characteristics of TB patients who were newly diagnosed versus previously diagnosed with HIV are compared. Genotypic clusters, defined as groups of two or more cases whose isolates of *Mycobacterium tuberculosis* had identical DNA fingerprints over the 10-year period or within 2 years of one another, were analyzed for their ability to predict HIV co-infection.

**Results:**

HIV testing rates were 26% before and 90% after the introduction of “opt-out” testing. During the “opt-out” testing years those <15 or >64 years of age at diagnosis were less likely to have been tested. In those tested the prevalence of HIV was 5.6%. In the age group 15–64 years, risk factors for HIV were: age (35–64 years), Canadian-born Aboriginal or foreign-born sub-Saharan African origin, and combined respiratory and non-respiratory disease. Compared to TB patients previously known to be HIV positive, TB patients newly discovered to be HIV positive had more advanced HIV disease (lower CD4 counts; higher viral loads) at diagnosis. Large cluster size was associated with Aboriginal ancestry. Cluster size predicted HIV co-infection in Aboriginal peoples when clusters included all cases reported over 10 years but not when clusters included cases reported within 2 years of one another.

**Conclusion:**

“Opt-out” HIV testing of TB patients is effective and well received. Universal HIV testing of TB patients (>80% of patients tested) has immediate (patients) and longer-term (TB/HIV program planning) benefits.

## Introduction

All tuberculosis (TB) patients ought to be tested for human immunodeficiency virus (HIV), but compliance with this recommendation has not yet been achieved in Canada or globally. In 2010 nationally, and in 2011 internationally, an HIV test result was reported in only 40% of TB patients [1], [2]. A lack of compliance was understandable before the availability of highly active anti-retroviral therapy (HAART) and in the context of discrimination, when informed consent and extensive pre- and post-test counseling were advised. More recently, however, these factors have been mitigated and both counseling and testing are considered part of routine medical care. Not knowing the HIV status of prevalent active TB cases has the potential to compromise TB management, outcomes and elimination [2], [3]. As such, poor compliance with the recommendation that all TB patients be HIV tested is now indefensible.

In response to two national advisories and the close biological and epidemiological links between HIV and TB, an ‘opt-out’ approach to HIV testing of TB patients was introduced in Alberta, a Province of Western Canada, in 2002–2003 [4], [5]. HIV testing is defined as ‘universal’ if >80% of incident TB cases were tested annually [6]–[8]. The ‘opt-out’ approach implemented in Alberta was patterned after an established program of ‘opt-out’ HIV testing of prenatal women [9], and is similar to the ‘provider-initiated’ HIV testing program recommended by the United Nations Programme on HIV/AIDS and World Health Organization (UNAIDS/WHO) in 2004 and 2007 for countries with high co-infection rates [10], [11]. This approach is characterized by two stages: 1) patients are briefly informed about the connection between HIV and TB and the clinical and prevention benefits of being tested and 2) the routine testing of TB patients for HIV unless their provider is actively informed of the patient's decision not to be tested.

We describe ten years of experience with universal ‘opt-out’ HIV testing of TB patients in Alberta. In addition to identifying risk factors for HIV seropositivity, the demographic, clinical and laboratory characteristics of TB patients who were newly diagnosed versus previously diagnosed with HIV are compared. Finally, we describe the extent to which clustering and/or cluster size, markers of recent transmission, predict HIV co-infection in the host using molecular epidemiologic techniques.

## Methods

Persons meeting the Canadian case definition for TB [1], and notified in the Province of Alberta (population 3,645,257 in 2011, Statistics Canada) over the 10-year period 2003 to 2012, were identified. In addition to HIV test results the following demographic and clinical information were abstracted from the Provincial TB Registry for each case: age, sex, population group, disease type (new active versus relapse/re-infection), first-line drug resistance, disease site, (respiratory, non-respiratory or both, according to the Canadian TB Standards) and mortality [1]. Population groups in this study include Canadian-born Aboriginal (First Nations [North American Indian], Métis or Inuit according to the Constitution Act of 1982), Canadian-born ‘other’, foreign-born from sub-Saharan Africa (as per the Canadian International Development Agency at: http://www.acdi-cida.gc.ca/subsaharanafrica) and foreign-born ‘other’. The proportion of all TB patients and TB patients aged 15 to 64 years who were HIV tested before and after the introduction of ‘opt-out’ testing, was related to the timing of two national advisories and the availability of HAART. All HIV testing of TB patients was performed in the Provincial Laboratory for Public Health (ProvLab) using conventional methods. Risk factors for HIV co-infection are described; in a multivariable model an *a priori* set of variables was retained.

For HIV co-infected patients data from individual health records supplements those data abstracted from the TB Registry (see below). HIV co-infected TB patients are divided into those who were known to be HIV-positive in the past and those who were discovered to be HIV positive during the 3 months immediately preceding their date of diagnosis of TB or during the period of active treatment of TB. The window for a “new positive” HIV test permits TB being unmasked or diagnosed during the early investigation and management of HIV [12]–[14]. TB patients with *known* positive HIV tests were compared to TB patients with *new* positive HIV tests on the basis of age, sex, population group, disease type, CD4 count, viral load (≤400 copies/ml – a viral load that is generally associated with controlled HIV infection on antiretroviral therapy [ARV's] and accounts for changing testing methodology; 401–100,000 copies/ml – moderately advanced HIV infection, and >100,000 copies/ml – the recognized cutoff for advanced HIV infection), hepatitis C serology, HIV exposure category and mortality. Results indicate whether TB patients were on ARVs at the time of diagnosis of TB.

Isolates of *M. tuberculosis* from all culture-positive cases of TB diagnosed in the Province of Alberta between 2003 and 2012 are routinely DNA fingerprinted using standardized restriction fragment-length polymorphism (RLFP), supplemented in those isolates with five or fewer copies of the insertion sequence *6110*, by spoligotyping [15], [16]. Images were digitalized using the imager video camera system (Appligene, Illkirch, France). Digitalized gel images were analyzed using Gelcompar II computer software (Applied Maths, Kortrijk, Belgium). The analysis was performed on coded specimens in a blinded fashion. All isolates matched as identical by computer were manually confirmed by visually comparing the original autoradiographs. ‘Clusters’ are defined as two or more case isolates with identical DNA fingerprints over either (i) the entire 10-year “opt-out” testing period or (ii) within two years of one another. For the latter we chronologically ordered the TB cases in each genotypic cluster by the date of diagnosis of each culture-positive case. We assumed that the first case of respiratory TB in a cluster resulted from reactivation of latent TB infection and was the source case patient. Subsequent respiratory and/or non-respiratory cases in the same genotypic cluster likely resulted from recent transmission and rapid progression to disease and were defined as secondary cases if they occurred within 24 months of the sourse case or each other [17], [18]. For each definition of a cluster, mean cluster size was compared by age, sex, population group, HIV status and population-group-by-HIV-status-interaction.

### Statistical Analysis

Fisher's exact test, where necessary, was used to compare proportions. To compare the demographic and clinical characteristics of HIV-negative and HIV-positive TB patients between 2003 and 2012, odds ratios (OR) with 95% confidence intervals were reported. Purposeful selection method was used to select the variables included in the multivariable logistic regression model [19]. Age, sex, population group, disease type and site, drug resistance, and mortality were tested for potential confounding effect. Wald's test was used to test the significance of each variable in the model. Generalized estimating equation (GEE) with identity link and independent correlation was used to account for clustering with the same strain, to estimate the mean cluster size after adjusting for age, sex, population group and HIV status. A p-value of 0.05 was used as statistically significant. All analyses were conducted using Stata (StataCorp. 2011. Stata Statistical Software: Release 12. College Station, TX: StataCorp LP). This study was approved by the Health Research Ethics Board of the University of Alberta. A waiver of written consent was provided as anonymous, routinely collected surveillance data were used.

## Results

**Figure 1:**
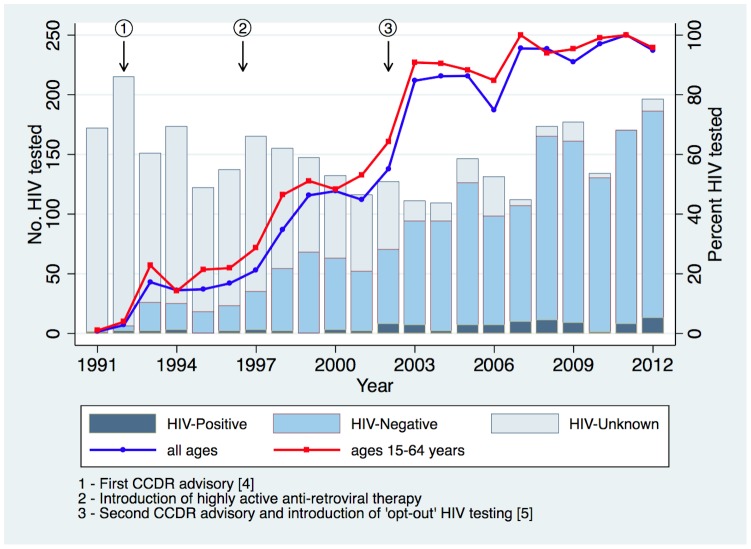
The absolute number of TB patients that were HIV tested (left axis) and the proportion of TB patients who were HIV tested (right axis) by year in Alberta, 1991–2012.

Between 1991 and 2002 the proportion of TB patients who were HIV tested averaged 26.1% (see Figure 1). In 1996–1997 the introduction of HAART and in 2002–2003 the introduction of “opt-out” HIV testing, resulted in sharp increases in the proportion of TB patients who were HIV tested. In 2003, and annually thereafter, “universal” HIV testing of TB patients was achieved (average 90.6%; range 74.8% to 100% overall and average 94.9%; range 84.8% to 100% in the age group 15–64 years). Of the 1317 TB patients who were HIV tested between 2003 and 2012, 74 (5.6%) were HIV-positive (see Table 1). Independent of sex or population group, TB patients in the age group 15–64 years were more likely to be HIV tested than TB patients in the age groups 0–14 years or >64 years (94.9% vs. 74.7%, p<0.001; and 94.9% vs. 81.3%, p<0.001, respectively). CBO were less likely to be HIV tested than other population groups but the difference was not statistically significant after adjustment for age. For patients who did not undergo HIV testing (n = 136), the test was not offered in 39 (28.7%), declined in 13 (9.6%), and in the remainder the reason for not testing was unknown. For patients who were not offered the test, 14 (35.9%) were diagnosed with TB at death and 16 (41.0%) were either younger than 15 or older than 64 years of age. For patients who declined HIV testing there was no consistent pattern in their demographic characteristics.

**Table 1:** HIV status of TB patients in Alberta by demographic group, 2003–2012.

Demographic group		TB Cases	HIV tested*	HIV Positive†
			n (%)	n (%)
Total		1453	1317 (90.6)	74 (5.6)
Age (years)				
	0–14	75	56 (74.7)	01 (1.8)
	15–34	458	443 (96.7)	21 (4.7)
	35–64	577	539 (93.4)	51 (9.5)
	>64	343	279 (81.3)	01 (0.4)
Sex				
	Male	748	685 (91.6)	45 (6.6)
	Female	705	632 (89.6)	29 (4.6)
Population group				
	CBO	154	130 (84.4)	01 (0.8)
	CBA	182	167 (91.8)	16 (9.6)
	FBO	854	771 (90.3)	15 (2.0)
	FBSSA	263	249 (94.7)	42 (16.9)

Abbreviations: CBO =  Canadian-born 'Other'; CBA =  Canadian-born Aboriginal; FBO = Foreign-born 'Other';

FBSSA = Foreign-born sub-Saharan African.

*Percent of all TB cases.

† Percent of HIV-tested TB cases.

Among HIV tested TB patients in the age group 15–64 years, 72/910 or 7.9% were HIV-positive (see Table 2). This seroprevalence was not significantly different from the one we reported in 1991-2002 (28/342 or 8.2%, p = 0.87) when much more selective testing was performed, suggesting, in the absence of major secular trends, that HIV co-infection may have been missed in the earlier era [7]. In the age group 15–64, those aged 35–64 years were more likely than those aged 15–34 years (9.5% vs. 4.7%, p<0.001) to be HIV co-infected in multivariate analysis (see Table 2). Similarly, Canadian-born Aboriginal or foreign-born sub-Saharan Africans were more likely than Canadian-born or foreign-born ‘other’ (12.5% vs. 2.2% and 18.3 vs. 2.2%, p<0.001 and p<0.001, respectively) to be HIV co-infected. Finally, patients with respiratory and non-respiratory TB combined were more likely than patients with respiratory TB alone (20.0% vs. 7.0%, p = 0.01), and patients reported as a TB death were more likely than patients not reported as a TB death (30.4% vs. 6.8%, p = 0.043) to be HIV co-infected. In contrast, HIV positive and negative TB patients did not differ by sex, disease type and presence of drug resistance.

**Table 2:** Risk Factors for HIV Seropositivity in TB patients aged 15–64 years, Alberta, 2003–2012.

Characteristic		HIV-negative	HIV-positive (%)	Univariate OR (95% CI; p-value)	Multivariate OR (95% CI; p-value)
		(N = 910)	(N = 72)		
Age (years)					
	15–34	422	21 (4.7)	1.00	1.00
	35–64	488	51 (9.5)	2.1 (1.24, 3.55; 0.006)	3.3 (1.83, 5.96; <0.001)
Sex*					
	Female	453	29 (6.0)	1.00	1.00
	Male	457	43 (8.6)	1.47 (0.9, 2.4; 0.12)	1.59 (0.94, 2.71; 0.09)
Population Group†					
	CBO	76	1 (1.3)	1.00	1.00
	FBO	535	13 (2.4)		
	CBA	112	16 (12.5)	6.23 (2.96, 13.13; <0.001)	5.42 (2.49, 11.82; <0.001)
	FBSSA	187	42 (18.3)	9.8 (5.24, 18.34; <0.001)	13.9 (7.14, 27.0; <0.001)
Disease Type					
	New Active	853	70 (7.6)	1.00	-
	Relapse/Retreatment	57	2 (3.4)	0.43 (0.1, 1.79; 0.19)	-
Drug Resistance‡					
	No	647	53 (7.6)	1.00	-
	Yes	119	10 (7.8)	1.02 (0.51, 2.07; 0.94)	-
	Unknown	144	9 (5.9)	-	-
Disease Site					
	Respiratory	545	41 (7.0)	1.00	1.00
	Non-respiratory	329	22 (6.3)	0.89 (0.52, 1.52; 0.6)	0.93 (0.52, 1.65; 0.8)
	Both	36	9 (20.0)	3.32 (1.5, 7.37; 0.003)	3.32 (1.29, 8.53; 0.013)
Mortality§					
	No	892	65 (6.8)	1.00	1.00
	Yes	18	7 (30.4)	6.02 (2.39, 15.15; <0.001)	2.80 (0.96, 8.15;0.059)

Abbreviations: CBO  =  Canadian-born 'Other'; CBA  =  Canadian-born Aboriginal; FBO  =  Foreign-born 'Other';

FBSSA  =  Foreign-born sub-Saharan African.

*By convention, sex was retained in the multivariable model. Its exclusion in the multivariable model did not affect the significance of other variables

† CBO and FBO were combined in the analysis as HIV prevalence rates were similarly low in both groups and the number of CBO cases was small

‡ Resistance to one or more first-line drug; cases whose drug resistance pattern was unknown were culture-negative.

§ Death before or during the period of active treatment of TB.

Of the 74 TB patients determined to be HIV co-infected in 2003-2012, 38 (51.4%) were known to have been HIV-positive prior to the diagnosis of TB and 36 (48.6%) were discovered to be HIV-positive at the time of diagnosis of TB (see Table 3). Those with known HIV positivity and those with new HIV positivity did not differ with respect to age, sex, population group, exposure category or mortality in univariate analysis. With respect to other characteristics, however, there were notable differences between groups. Compared to known HIV-positive cases, new HIV-positive cases were more likely to have respiratory TB in combination with non-respiratory TB, a low CD4 count (<200×10^6^/L) and a high viral load (>100,000 HIV RNA copies/ml); odds ratios and 95% confidence intervals 2.71(1.04, 6.99), 7.65(1.92, 30.5), and 12.7(1.32, 121.5), respectively. In patients with an ARV utilization history, 14 of 34 (41.2%) with known HIV positivity and none of 31 (0.0%) with new HIV positivity, were on ARV's at the time of diagnosis of TB (see Figure S1). TB patients with new HIV positivity were less likely than TB patients with known HIV positivity to be HCV positive, 8.6% vs 28.6% (0.23; 0.06, 0.94). In data that is not shown, HCV positivity was strongly correlated with IDU, 11 of 13 (84.6%) with IDU versus 0 of 36 (0.0%) without IDU, were HCV positive (p<0.0001). And IDU was strongly correlated with population group; of the 53 HIV positive TB patients whose exposure category was known, 11/14 (78.6%) Aboriginal peoples versus 2/39 (5.1%) persons of other population groups, reported IDU (p<0.001).

**Table 3:** Demographic and clinical characteristics of HIV co-infected TB patients according to the date of diagnosis of HIV relative to the date of diagnosis of TB*.

Characteristics		Total	Known HIV(+ve)*	New HIV(+ve)*	Odds ratio (95% CI; p-value)
		n	n (%)	n (%)	
No. assessed (%)		74	38	36	-
Age (years)					
	<35	22	14 (36.8)	08 (22.2)	0.49 (0.17, 1.36, 0.17)
	≥35	52	24 (63.2)	28 (77.8)	1.00
Sex					
	Female	29	18 (47.4)	11 (30.6)	1.00
	Male	45	20 (52.6)	25 (69.4)	2.04 (0.79, 5.31, 0.14)
Population group					
	CBO	01	01 (2.6)	0 (0.0)	Included with FBO
	CBA	16	10 (26.3)	06 (16.7)	0.36 (0.09, 1.51, 0.16)
	FBO	15	05 (13.2)	10 (27.8)	1.00
	FBSSA	42	22 (57.9)	20 (55.6)	0.54 (0.17, 1.77, 0.31)
Disease site					
	Respiratory	42	26 (68.4)	16 (44.4)	1.00
	Non-Respiratory	23	09 (23.7)	14 (38.9)	2.71 (1.04, 6.99, 0.04)
	Both	09	03 (7.9)	06 (16.7)	Included with Non-Resp
CD4 count †					
	<200	43	17 (44.7)	26 (72.2)	7.65 (1.92, 30.5, 0.004)
	≥200	18	15 (39.5)	03 (8.3)	1.00
	Unknown	13	06 (15.8)	07 (19.4)	-
HIV RNA (copies/ml) ‡					
	≤400	7	06 (15.8)	01 (2.8)	1.00
	401–100,000	21	14 (36.8)	7 (19.4)	3.0 (0.30, 30.0, 0.35)
	>100,000	28	09 (23.7)	19 (52.8)	12.7 (1.32, 121.5, 0.03)
	Unknown	18	09 (23.7)	09 (25.0)	-
Hepatitis C					
	Pos	13	10 (28.6)	03 (8.6)	0.23 (0.06, 0.94, 0.04)
	Neg	57	25 (71.4)	32 (91.4)	1.00
	Unknown	04	03 (7.9)	01 (2.8)	-
Exposure category §					
	HSS	36	17 (44.7)	19 (52.8)	1.00
	IDU	13	10 (26.3)	03 (8.3)	0.27 (0.06, 1.14, 0.07)
	Other	04	03 (7.9)	01 (2.8)	0.30 (0.03, 3.14, 0.31)
	Unknown	21	08 (21.1)	13 (36.1)	
Mortality					
	No death	66	33 (86.8)	33 (91.7)	1.00
	Died	08	05 (13.2)	03 (8.3)	0.6 (0.13, 2.72, 0.51)

abbreviations: CBO Canadian-born 'other'; CBA Canadian-born Aboriginal; FBO foreign-born 'other'; FBSSA foreign-born sub-Saharan African; IDU injection drug use; HSS heterosexual sex.

*The date of diagnosis of TB is defined as the start date of treatment; see text for definition of “known” vs. “new” HIV-positive

† Refers to the number of CD4 receptor-bearing lymphocytes ×10^6^/L; only measurements made up to three months before or one month after the start date of treatment of TB were accepted. If multiple measurements were made in this time period the measurement closest to the date of diagnosis of TB was used.

‡ Quantitative HIV viral loads were assessed in the Provincial Laboratory for Public Health; only measurements made up to three months before or one month after the start date of treatment of TB were accepted. If multiple measurements were made in this time period the measurement closest to the date of diagnosis of TB was used.

§ 2 HSS also listed ‘transfusion’; 10 IDU also listed 'heterosexual'; ‘other’ exposure categories included 2 men who have sex with men, and 2 vertical exposure.

**Figure 2:**
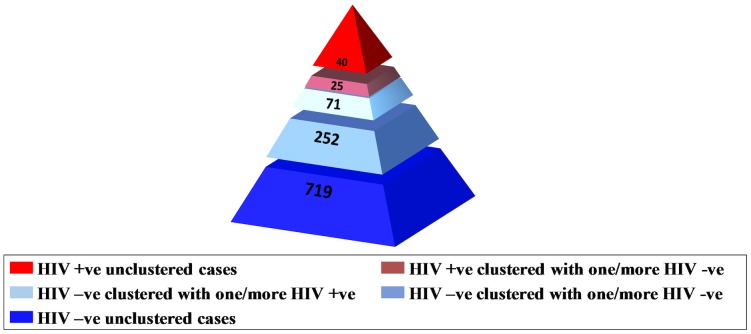
TB patients in Alberta who were culture-positive for *Mycobacterium tuberculosis*, whose isolates were DNA fingerprinted, and who were HIV tested (n = 1107) by clustering.

Of the 1453 patients diagnosed with TB between 2003 and 2012, 228 (15.7%) were culture-negative, 18 (1.2%) were culture-positive for MTB complex species other than *M. tuberculosis* and 1207 (83.1%) were culture-positive for *M. tuberculosis* (see Figure S2). HIV co-infection varied by culture status; 5/228 (2.2%) if culture-negative, 3/18 (16.7%) if culture-positive for MTB complex species other than *M. tuberculosis* and 65/1207 (5.4%) if culture-positive for *M. tuberculosis*. Of the *M*. *tuberculosis* isolates, 1202 (99.7%) were DNA fingerprinted (65 [5.4%] from HIV-positive patients, 1042 [86.7%] from HIV-negative patients and 95 [7.9%] from HIV-unknown patients). Of the 1107 HIV tested cases with DNA fingerprinted isolates 759 cases exhibited unique fingerprint patterns (non-clustered cases) and 348 (31.4%) clustered cases were dispersed among 104 clusters, when the analysis of clustering was unconstrained by time; i.e. any genotypic match over 10 years was accepted. Eighteen clusters (96 cases) involved one or more HIV-positive case (median cluster size 3.5 [2]–[16] cases); 86 clusters (252 cases) involved HIV-negative cases only (median cluster size 2.0 [2]–[18] cases), p<0.03 (see Figure 2). Only one cluster involved both CBA and FBSSA cases. Using the same unconstrained cluster analysis mean cluster size did not differ by age, sex or HIV status (see Table 4). Mean cluster size was, however, much larger in Aboriginal peoples compared to other population groups, and within Aboriginal peoples, larger in those who were HIV-positive compared to those who were HIV-negative. When the cluster analysis was constrained by time (i.e. clustered cases had to have occurred within 24 months of one another [see Methods], thus better reflecting recent transmission, cluster size was again much larger in Aboriginal peoples, but within Aboriginal peoples an HIV interactive effect was no longer seen.

**Table 4:** Mean cluster size by demographic group, HIV status and population group-HIV status interaction in Alberta, 2003–2012.

		Clustering with no time frame		Clustering with 2-yr time frame	
Characteristics		Cluster size	Overall	Cluster size	Overall
		Mean (SE)	Significance*	Mean (SE)	Significance*
Age			0.23		0.45
	<15 years	3.50 (0.56)		2.7 (0.5)	
	15–34 years	2.58 (0.16)		2.08 (0.15)	
	35–64 years	2.63 (0.15)		2.1 (0.13)	
	>64 years	2.33 (0.21)		1.89 (0.18)	
Sex			0.15		0.13
	Male	2.69 (0.13)		2.18 (0.12)	
	Female	2.43 (0.14)		1.93 (0.12)	
Population group			<0.0001		<0.0001
	CBA	6.88 (0.25)		5.38 (0.23)	
	CBO & FBO	1.82 (0.12)		1.49 (0.11)	
	FBSSA	1.98 (0.23)		1.61 (0.2)	
HIV Status			0.54		0.51
	HIV-negative	2.57 (0.10)		2.07 (0.09)	
	HIV-positive	2.38 (0.58)		1.82 (0.52)	
Population group by HIV-status interaction			0.017		0.11
	CBA HIV-negative	6.76 (0.26)		5.3 (0.24)	
	CBA HIV-positive	8.81 (0.78)		6.58 (0.7)	
	CBO & FBO HIV-negative	1.85 (0.12)		1.52 (0.1)	
	CBO & FBO HIV-positive	1.34 (0.83)		1.02 (0.75)	
	FBSSA HIV-negative	2.02 (0.24)		1.64 (0.21)	
	FBSSA HIV-positive	1.34 (0.53)		1.15 (0.47)	

Abbreviations: CBA Canadian-born Aboriginal; CBO Canadian-born ‘other’; FBO foreign-born ‘other’; FBSSA foreign-born sub-Saharan African.

*Significance of difference between categories obtain from Wald chi-squared test in multivariate analysis.

## Discussion

In the time period and jurisdiction of this study, the routine ‘opt-out’ HIV testing of TB patients was very well received, with only 9.4% of incident TB cases not being tested. Similar to experience in the United States, TB patients at the extremes of age (<15 and >64 years) were less likely to be tested [20]. In those who were HIV tested the prevalence of HIV was 5.6%, almost identical to the rate estimate made for Canada by the WHO in 2007 (5.7%) [21]. In the age group 15–64 years, which included 97.3% of the co-infected patients, HIV was more common in those aged 35–64 years, Canadian-born Aboriginal or foreign-born sub-Saharan African (HIV prevalence's 12.5% and 18.3%, respectively), those with combined respiratory and non-respiratory TB, and those who died. Compared to patients with known HIV positivity, patients with new HIV positivity at the time of diagnosis of TB, had more advanced HIV disease (lower CD4 counts; higher viral loads). Median cluster size was greater when clusters contained one or more HIV-positive case than when clusters contained HIV-negative cases only. Mean cluster size was greater in HIV-positive versus HIV-negative Aboriginal peoples.

In the WHO's package of collaborative TB/HIV activities aimed at reducing the burden of TB/HIV, TB patients are recommended to be HIV tested [22], [23]. Quite appropriately, UN/AIDS/WHO pronouncements on provider-initiated (‘opt-out’) HIV counseling and testing of TB patients have focused on countries with high co-infection rates where the potential return on HIV testing of TB patients is greatest in terms of preventing morbidity, mortality and sustained transmission [10], [11], [24]. Despite some difficulties in implementation such as the structural and personal factors described by Pope et al. in South Africa [25], [26], a remarkable 69% of TB patients in the African Region had a documented HIV test result in 2011 [2]. In high income countries such as Canada co-infection rates are lower, limiting the potential impact of screening, but the resources necessary to maximize the clinical and prevention benefits of screening are more readily available. Since 1989 in the US and 1992 in Canada, national advisories have recommended universal HIV testing of TB patients [4], [5], [27]. In Alberta, the availability of ART in 1996–1997 and the introduction of ‘opt-out’ HIV testing in 2002–03, the latter facilitated by centralized clinical and laboratory TB services [28], resulted in universal HIV testing of TB patients. Though the prevalence of HIV co-infection was low at the extremes of age and in those who were Canadian-born non-Aboriginal or foreign-born from countries other than those in sub-Saharan Africa, rates in all of these groups were still >0.1%, the rate above which testing is considered cost-effective [29], [30]. Nationally, it is unknown whether the low proportion of TB patients with a reported HIV test result is due to a failure to record the test result, to offer the test, or to the test being declined, once offered [1]. Differences in the organization of TB programs in Canada's provinces and territories and perhaps some under-appreciation of TB as a clinical opportunity for HIV testing [31], may otherwise explain the low proportion of TB patients that are HIV tested in Canada.

Universal HIV testing of TB patients allowed us to further refine our understanding of the risk factors for co-infection of TB patients in Alberta and by extrapolation, other major immigrant-receiving provinces of Canada. As in earlier analyses, age and population group emerged as significant risk factors [7], [8]. Higher HIV co-infection rates in middle-aged versus young adult TB patients is understood to reflect not only age at HIV acquisition but more immunologically advanced disease and therefore higher risk of TB associated with years of living with HIV (see below). The latest recommendations of the CDC and the US Preventive Services Task Force, which are in turn based upon evidence that earlier initiation of ART (when CD4 counts are between 200 and 500×10^6^/L), is associated with reduced risk for AIDS-related events or deaths [30], [32], suggests that broader, more inclusive testing is the direction Canada should be taking. In this regard, the US has for some time now recommended HIV testing of TB contacts [33]; more recently this has been shown to be feasible, using either conventional or point-of-care testing methods [34], [35].

The high HIV prevalence in Aboriginal TB patients is especially troubling; Aboriginal peoples in Canada are known to be at higher risk for latent TB infection and the penetration of HIV into this subpopulation has the potential to aggravate what is already an unsatisfactory state of TB control [1]. Though HIV testing is part of the immigration medical examination in Canada, it is unlikely to result in the exclusion of the applicant [36]. The high HIV co-infection rate in immigrants to Alberta from sub-Saharan Africa with TB is not altogether unexpected [24]. The prevalence does, however, add weight to the argument that all immigrants and refugees with HIV infection ought to be referred for medical surveillance for both HIV and TB as a condition of entry to Canada. That TB patients with combined respiratory and non-respiratory TB or TB patients that died, were more likely to be HIV co-infected was not surprising [1], [7], [20].

Though the number of HIV co-infected TB patients was relatively small, representing a potential study limitation, a new diagnosis of HIV was just as common as a known diagnosis of HIV. Within the limitations of the data (small numbers; missing data) those with a new diagnosis of HIV and therefore without ART, were as expected, more likely to be immunosuppressed and to have both respiratory and non-respiratory disease combined. This notwithstanding, many of those known to have been HIV positive in the past were immunosuppressed and not on ARVs at the time of diagnosis of TB. The reasons for this and the possibility that TB may have been prevented in these patients and those with a new diagnosis of HIV, had they been HIV tested earlier as a TB contact, will be explored in a separate communication. Screening persons with HIV for TB at regular intervals, allowing for earlier diagnosis and treatment of TB, might lower mortality [37]. Diagnosing HIV in contacts followed by treatment with ART and TB preventive therapy might prevent TB [38]. As expected, HCV was closely associated with IDU, placing this subpopulation of the HIV co-infected at greater risk of anti-TB drug induced liver injury [39]. That IDU was commonly reported in HIV-positive Aboriginal TB patients suggests that efforts to prevent IDU-related HIV infection might also prevent TB in this population group.

In earlier studies HIV was shown to increase the risk of belonging to a cluster of TB cases [40], [41], but not to affect the size of clusters [42]. More recently, in a study from San Francisco, median cluster size was shown to be larger when clusters involved one or more HIV-positive case than when clusters involved HIV-negative cases only; within the homeless population HIV was judged to be a key factor sustaining TB transmission [17], [43]. In Alberta, where being of Aboriginal ancestry is well known to be associated with clustering [44], cluster size was much larger in Aboriginal peoples, but within Aboriginal peoples there was no HIV interactive effect. In earlier studies in Alberta no association between Beijing strains and either transmission or HIV was found[18], [45]. In contrast to HIV endemic countries, where most TB in HIV-positive persons is thought to result from recent transmission [46], the large proportion of ‘un-clustered’ HIV-positive patients (almost all foreign-born) in our study suggests that reactivation TB is the predominate pathogenic mechanism for HIV-related TB in high income immigrant-receiving countries. Dual control measures would appear to be indicated based on population groups, emphasizing prevention of transmission in Aboriginal peoples and treatment of latent infection in the foreign-born.

## Supporting Information

Figure S1 **Anti-retroviral (ARV) utilization history of HIV co-infected TB patients according to date of diagnosis of HIV.**
(TIF)

Figure S2 **Culture Status and HIV status of TB Patients in Alberta, 2003–2012.**
(TIF)

## References

[1] The Canadian Tuberculosis Standards, 7th Edition. Menzies D, Ed. (2013) Can Respir J 20(Suppl A): 1A–174A. 10.14745/ccdr.v40i06a04 PMC5864480 29769892

[2] World Health Organization. Global Tuberculosis Report 2012. Available: http://apps.who.int/iris/bitstream/10665/75938/1/9789241564502_eng.pdf. Accessed: 2014 May 14

[3] UNAIDS Report on the Global AIDS Epidemic 2012. Global Report. Available: http://www.unaids.org/en/media/unaids/contentassets/documents/epidemiology/2012/gr2012/20121120_unaids_global_report_2012_with_annexes_en.pdf http://www.unaids.org/en/media/unaids/contentassets/documents/epidemiology/2012/gr2012/20121120_unaids_global_report_2012_with_annexes_en.pdf

[4] Canadian Thoracic Society, Tuberculosis Directors of Canada, Department of National Health and Welfare in consultation with the provincial and territorial epidemiologists, AIDS coordinators and HIV caregivers (1992) Guidelines for the identification, investigation and treatment of individuals with concomitant tuberculosis and HIV infection. CCDR 18: 155–160. 1291007

[5] The Canadian Tuberculosis Committee of the Centre for Infectious Disease Prevention and Control, Population and Public Health Branch, Health Canada (2002) Recommendations for screening and prevention of tuberculosis in patients with HIV and for screening for HIV in patients with tuberculosis and in their contacts. CCDR 28(ACS-7): 1–6. 12501746

[6] Horsburgh C, Feldman S, Ridzon R (2000) Practice Guidelines for the treatment of tuberculosis. CID 31: 633–639. 10.1086/314007 11017808

[7] Sturtevant D, Preiksaitis J, Singh A, Houston S, Gill J, et al (2009) The feasibility of using an ‘opt-out’ approach to achieve universal HIV testing of tuberculosis patients in Alberta. Can J Public Health 100: 116–120. 19839287 10.1007/BF03405519 PMC6974056

[8] Long R, Boffa J (2010) High HIV-TB co-infection rates in marginalized population: evidence from Alberta in support of screening TB patients for HIV. Can J Public Health 101: 202–204. 20737809 10.1007/BF03404374 PMC6973978

[9] Jayaraman G, Preiksaitis J, Larke B (2003) Mandatory reporting of HIV infection and opt-out prenatal screening for HIV infection: Effect on testing rates. CMAJ 168: 679–682. 12642422 PMC154912

[10] UNAIDS/WHO (2004) Policy statement on HIV testing. Available: http://data.unaids.org/una-docs/hivtestingpolicy_en.pdf. Accessed: 2014 May 14

[11] WHO/UNAIDS (2007) Guidance on provider-initiated HIV testing and counseling in health facilities. Available: http://whqlibdoc.who.int/publications/2012/9789241503006_eng.pdf. Accessed: 2014 May 14

[12] Girardi E, Sabin CA, Monforte A d'A, Hogg B, Phillips AN, et al (2005) Incidence of tuberculosis among HIV-infected patients receiving highly active antiretroviral therapy in Europe and North American. CID 41: 1772–1782. 10.1086/498315 16288403

[13] Brinkhof MW, Egger M, Boulle A, May M, Hosseinpour M, et al (2007) Tuberculosis after initiation of antiretroviral therapy in low-income and high-income countries. CID 45: 1518–1521. 10.1086/522986 PMC3687541 17990236

[14] Manabe YC, Breen R, Perti T, Girardi E, Sterling TR (2009) Unmasked tuberculosis and tuberculosis immune reconstitution inflammatory disease: a disease spectrum after initiation of antiretroviral therapy. J Infect Dis 199: 437–444. 19090776 10.1086/595985

[15] Van Embden JDA, Cave MD, Crawford JT, Dale JW, Eisenach KD, et al (1993) Strain identification of *Mycobacterium tuberculosis* by DNA fingerprinting: recommendations for a standardized methodology. J Clin Microbiol 31: 406–409. 8381814 10.1128/jcm.31.2.406-409.1993 PMC262774

[16] Dale JW, Brittain D, Cataldi AA, Cousins D, Crawford JT, et al (2001) Spacer oligonucleotide typing of bacteria of the *Mycobacterium tuberculosis* complex: recommendations for standardized nomenclature. Int J Tuberc Lung Dis 5: 216–219. 11326819

[17] DeRiemer K, Kawamura LM, Hopewell PC, Daley CL (2007) Quantitative impact of human immunodeficiency virus infection on tuberculosis dynamics. Am J Respir Crit Care Med 176: 936–944. 17690336 10.1164/rccm.200603-440OC PMC2048673

[18] Langlois-Klassen D, Senthilselvan A, Chui L, Kunimoto D, Saunders D, et al (2013) Transmission of *Mycobacterium tuberculosis* Beijing strains, Alberta, Canada, 1991–2007. Emerg Infect Dis 19: 701–711. 23648234 10.3201/eid1905.121578 PMC3649004

[19] Hosmer DW Jr, Lemeshow S (2004) Applied Logistic Regression. 2^nd^ Ed. John Wiley and Sons, Toronto, 2004.

[20] Centre for Disease Control (CDC) (2010) Mortality among patients with tuberculosis and association with HIV status-United States, 1993–2008. MMWR 59: 1509–1513. 21102404

[21] World Health Organization (2009) Global tuberculosis control, 2009: epidemiology, strategy, financing. Geneva: World Health Organization. WHO/HTM/TB/2009.411

[22] Policy on collaborative TB/HIV activities (2010) Geneva. World Health Organization, 2004 (WHO/HTM/TB/2004.330;WHO/HTM/HIV/2004.1)

[23] World Health Organization (2012) WHO policy on collaborative TB/HIV activities: guidelines for national programmes and other stakeholders. Geneva, 2012 (WHO/HTM/TB/2012.1). 23586124

[24] Chaisson RE, Martinson NA (2008) Tuberculosis in Africa-Combating an HIV-driven crisis. N Engl J Med 358: 1089–1092. 18337598 10.1056/NEJMp0800809

[25] Pope DS, Atkins S, DeLuca AN, Hausler H, Hoosain E, et al (2010) South African TB nurses' experiences of provider-initiated HIV counseling and testing in the Eastern Cape Province: a qualitative study. AIDS Care 22: 238–245. 20390502 10.1080/09540120903040594

[26] Pope DS, DeLuca AN, Kali P, Hausler H, Sheard C, et al (2008) A cluster randomized trial of provider initiated (Opt-out) HIV counseling and testing of tuberculosis patients in South Africa. J Acquir Immune Defic Syndr 48: 190–195. 18520677 10.1097/QAI.0b013e3181775926 PMC2632747

[27] Centre for Disease Control (CDC) (1989) Tuberculosis and immunodeficiency virus infection: Recommendations of the Advisory Committee for the Elimination of Tuberculosis (ACET). MMWR 38(14): 236–238, 243–250. 2494425

[28] Jensen M, Lau A, Langlois-Klassen D, Boffa J, Manfreda J, et al (2012) Eliminating tuberculosis: a population-based study of TB epidemiology and innovative service delivery in Canada. Int J Tuberc Lung Dis 16: 43–49. 22236844 10.5588/ijtld.11.0374

[29] Sanders GE, Bayoumi AM, Holodniy M, Owens DK (2008) Cost-effectiveness of HIV screening in patients older than 55 years of age. Ann Intern Med 148: 889–903. 18559840 10.7326/0003-4819-148-12-200806170-00002 PMC3428219

[30] Moyer VA, on behalf of the U.S. Preventive Services Task Force (2013) Screening for HIV: U.S. Preventive Services Task Force Recommendation Statement. Ann Intern Med PMID: 23698471.

[31] Long R. Langlois-Klassen D (2013) A clinical opportunity for routine HIV testing. CMAJ 185: 587. 10.1503/cmaj.113-2113 PMC3626813 23589539

[32] Branson BM, Handsfield HH, Lampe MA, Janssen RS, Taylor AW, et al (2006) Revised recommendations for HIV testing of adults, adolescents, and pregnant women in health-care settings. MMWR Recomm Rep 55: 1–17. 16988643

[33] Centers for Disease Control (1994) Revised Guidelines for HIV Counseling, Testing and Referral. MMWR 50 (RR-19): 1–58. 11718472

[34] Gardner A, Naureckas C, Beckwith C, Losikoff P, Martin C, et al (2012) Experiences in implementation of routine human immunodeficiency virus testing in a US tuberculosis clinic. Int J Tuberc Lung Dis 16: 1241–1246. 22793872 10.5588/ijtld.11.0628

[35] Person AK, Goswami ND, Bissett DJ, Turner DS, Baker AV, et al (2010) Pairing QuantiFERON TB Gold In-Tube with opt-out HIV testing in a tuberculosis contact investigation in the SouthEastern United States. AIDS Patient Care and STDs 24: 539–546. 20731612 10.1089/apc.2010.0102 PMC2958451

[36] Zencovich M, Kennedy K, MacPherson DW, Gushulak BD (2006) Immigration medical screening and HIV infection in Canada. Int J STD and AIDS 17: 813–16. 17212857 10.1258/095646206779307469

[37] Reid A, Scano F, Getahun H (2006) Towards universal access to HIV prevention, treatment, care and support: the role of tuberculosis/HIV collaboration. Lancet Infect Dis 6: 483–495. 16870527 10.1016/S1473-3099(06)70549-7

[38] Akolo C, Adetifa I, Shepperd S, Volmink J (2010) Treatment of latent tuberculosis infection in HIV infected persons. Cochrane Database Syst Rev Jan 20: CD000171. 10.1002/14651858.CD000171.pub3 PMC7043303 20091503

[39] Staples CT, Rimland D, Dudas D (1999) Hepatitis C in the HIV (Human Immunodeficiency Virus) Atlanta V.A. (Veterans Affairs Medical Center) Cohort Study (HAVACS): The Effect of Coinfection on Survival. CID 29: 150–154. 10.1086/520144 10433578

[40] Alland D, Kalkut GE, Moss AR, McAdam RA, Hahn JA, et al (1994) Transmission of tuberculosis in New York city: an analysis by DNA fingerprinting and conventional epidemiological methods. N Engl J Med 330: 1710–1716. 7993412 10.1056/NEJM199406163302403

[41] Jasmer RM, Hahn JA, Small PM, Daley CL, Behr MA, et al (1999) A molecular epidemiological analysis of tuberculosis trends in San Francisco, 1991-1997. Ann Intern Med 130: 971–978. 10383367 10.7326/0003-4819-130-12-199906150-00004

[42] Giordano T, Soini H, Teeter L, Adams G, Musser J, et al (2004) Relating the size of molecularly defined clusters of tuberculosis to the duration of symptoms. CID 38: 10–6. 10.1086/380454 14679442

[43] Mohtashemi M, Kawamura LM (2010) Empirical evidence for synchrony in the evolution of TB cases and HIV + contacts among the San Francisco homeless. PloS One 5: e 8851. 10.1371/journal.pone.0008851 PMC2809753 20107514

[44] Kunimoto D, Sutherland K, Manfreda J, Wooldrage K, Fanning A, et al (2004) Transmission characteristics of tuberculosis in the foreign–born and Canadian-born population of Alberta, Canada. Int J Tuberc Lung Dis 8: 1213–1220. 15527153

[45] Langlois-Klassen D, Kunimoto D, Saunders LD, Chui L, Boffa J, et al (2013) A Population based cohort study of *Mycobacterium tuberculosis* Beijing strains: An emerging public health threat in an immigrant-receiving country? PLoS One 7: e38431 doi:10.1371/journal. pone. ee38431 10.1371/journal.pone.0038431 PMC3367965 22679504

[46] Houben RMGJ, Crampin AC, Ndhlovu R, Sonnenberg P, Godfrey-Faussett P, et al (2011) Human immunodeficiency virus associated tuberculosis more often due to recent infection than reactivation of latent infection. Int J Tuberc Lung Dis 15: 24–31. 21276292

