# Supplementary figures and images for: A 10-Year Population Based Study of ‘Opt-Out’ HIV Testing of Tuberculosis Patients in Alberta, Canada: National Implications
Source: PLoS One. 2014 Jun 9;9(6):e98993. doi: 10.1371/journal.pone.0098993 (PMC4049754; doi:10.1371/journal.pone.0098993)

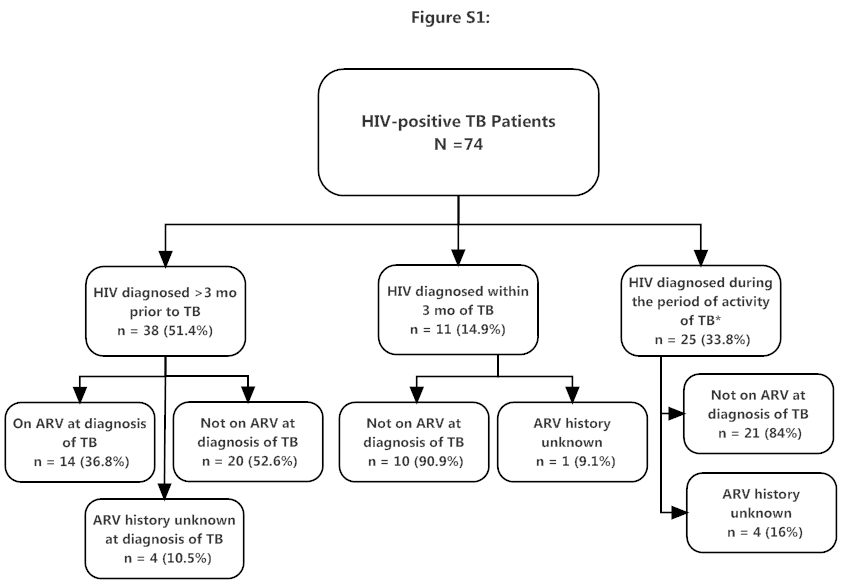

Supplement: Figure S1 — Anti-retroviral (ARV) utilization history of HIV co-infected TB patients according to date of diagnosis of HIV. (TIF) [file pone.0098993.s001.tif]

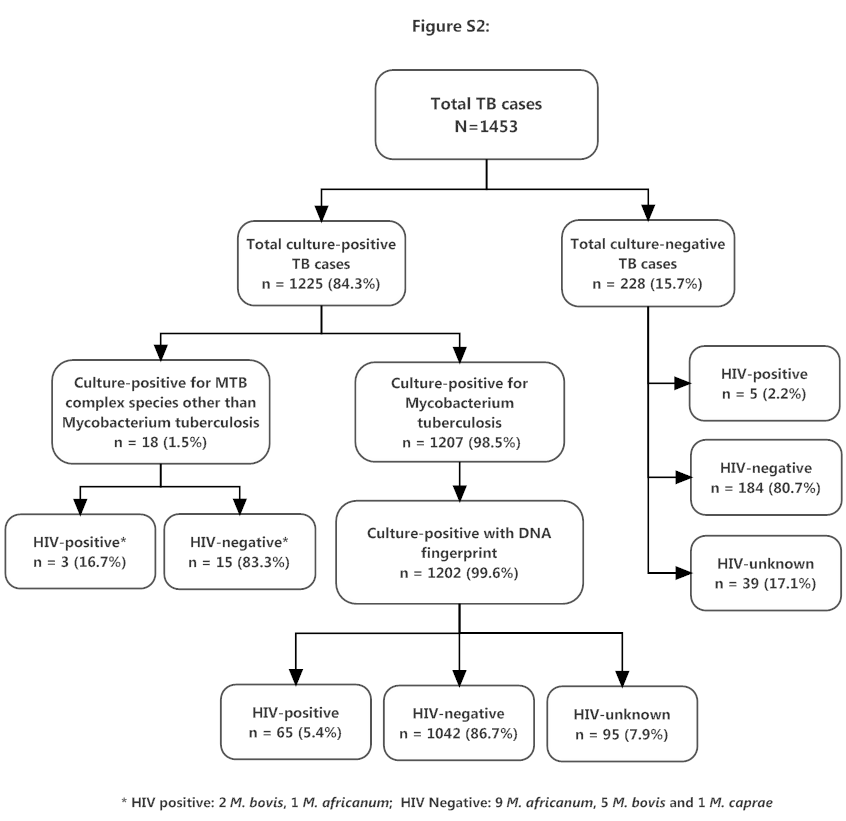

Supplement: Figure S2 — Culture Status and HIV status of TB Patients in Alberta, 2003–2012. (TIF) [file pone.0098993.s002.tif]
